# Knowledge, Attitudes, and Practices among Healthcare Workers regarding Depression Care in Two Medium-Sized Hospitals in Kenya

**DOI:** 10.1155/2024/4756962

**Published:** 2024-06-07

**Authors:** Millicent Muthoni Muriuki, Peterson Mwangi, Ezra Kombo Osoro, Miriam Miima

**Affiliations:** ^1^School of Humanities and Social Sciences, United States International University-Africa, Nairobi, Kenya; ^2^Mental Health Unit, AIC Kijabe Hospital, Kijabe, Kenya; ^3^School of Medicine, Masinde Muliro University of Science and Technology, Kakamega, Kenya

## Abstract

**Introduction:**

Depression is the most common mental health disorder worldwide with a lifetime prevalence of approximately 10% in the general population. Our objective was to assess the knowledge, attitudes, and practices among healthcare workers (HCWs) regarding depression care.

**Methods:**

We conducted a cross-sectional study among consenting healthcare workers in two medium-sized hospitals in Kenya. Data on demographic characteristics, knowledge, attitude, and practice of depression were collected through a self-administered structured questionnaire. The Revised Depression Attitude Questionnaire was incorporated into the questionnaire. Knowledge and attitude scores were computed, where higher scores suggested higher knowledge or more positive attitudes. Descriptive and regression analyses were used to assess associations, and a *p* value of < 0.05 was considered significant.

**Results:**

Among the 316 HCWs approached, 303 (95.9%) consented and were enrolled. Almost two-thirds (64.0%) of the respondents were female, and 58.4% were between 18 and 29 years old. HCWs were categorised into three: nurses, clinicians (doctors/clinical officers), and nonclinicians (other healthcare workers). The median knowledge score among respondents was 9 out of 10. Nonclinicians scored significantly lower (*β* = −0.5, *p* < 0.011) on the knowledge score compared to clinicians. Only 9.3% of the respondents strongly agreed or agreed that they were confident in assessing the risk of suicide in patients with depression. The median attitude score among respondents was 65 out of 110. The attitude score was positively associated with the knowledge score (*β* = 0.78, *p* = 0.001), and respondents with professional experience of 5-14 years had higher attitude scores compared (*β* = 1.7, *p* = 0.023) to those with fewer than 5 years. Among clinicians and nurses, 40.3% reported that they rarely or have never been screened for depression.

**Conclusions:**

HCWs demonstrated good knowledge of depression's symptoms and causes but lacked confidence in pharmacological management, with gaps in regular screening and comprehensive care practices, particularly among nonclinicians and less experienced staff. Focused training for these groups could enhance the early detection and treatment of depressed patients.

## 1. Introduction

Depression, affecting about 10% of the global population and up to 20% in clinical settings, poses significant public health challenges [1]. It is characterized by persistent sadness and loss of interest and can lead to serious outcomes including dementia and suicide [2]. In Kenya, studies among specific groups reported a depression prevalence of 32% [3, 4].

Diagnosing major depressive disorder (MDD) is challenging due to its overlap with mental disorders like anxiety and physical illnesses, leading to frequent underdiagnosis [5]. Additionally, stigma in healthcare settings, often fueled by healthcare workers' negative attitudes towards mental health, exacerbates these challenges and affects patient care [6].

Kenya is a low- to middle-income country with a considerable mental health treatment gap and where few healthcare workers have advanced training in mental health. Therefore, the Kenya Mental Health Plan of Action (2021-2025) has provided that healthcare providers (HCP) at different levels will need to participate in mental health provision (detection, treatment, and follow-up) as a way of addressing the large treatment gap for mental illnesses [7, 8]. However, there is limited knowledge about the readiness and willingness of primary care workers to undertake these roles. Understanding their knowledge, attitudes, and practices (KAP) is crucial for this initiative.

Research on the KAP of healthcare workers towards depression in Kenya is limited. Studies show that healthcare workers have moderate to high knowledge but find managing depression challenging. A survey across Kenya's provinces revealed a reluctance to admit mental illness patients to medical wards [9]. Additionally, a recent study found that 90% of healthcare workers lacked specific mental health training in the past five years [10], potentially leading to delayed depression diagnosis and treatment.

Understanding the KAP of healthcare workers regarding depression is crucial for enhancing clinical care and informing policy, as outlined in the Kenya Mental Health Action Plan (2021-2025). This is especially relevant in regions with limited specialized mental healthcare. Here, we assessed KAP regarding depression care among healthcare staff in two medium-sized health facilities in Kenya.

## 2. Materials and Methods

### 2.1. Study Design and Sites

We conducted a cross-sectional study in two health facilities in Kenya—AIC Kijabe Mission and Naivasha County Referral Hospitals—between December 2021 and February 2022. Kijabe Mission Hospital is a faith-based 300-bed facility, while Naivasha County Referral Hospital is a 250-bed public hospital [11]. The number of healthcare workers in the two facilities is estimated at 450 and 270, respectively. The two facilities offer secondary- to tertiary-level medical care, with Kijabe Hospital being classified as a level 6 facility, while Naivasha is classified as a level 4 facility [12]. At the time of the study, Naivasha Hospital had dedicated mental health unit staff, while in Kijabe, mental healthcare was integrated into the medical department. Conducting the study in a public and faith-based institution offered the opportunity to measure knowledge, attitude, and practice about depression among healthcare workers in different workplace settings.

The study population was healthcare workers working in the selected health facilities. Healthcare workers were defined as staff who were involved in the provision of clinical care in the study facility. Healthcare workers who consented to participate in the study were enrolled.

### 2.2. Sample Size and Sampling

The sample size was estimated with an assumption of the standard deviation of the attitude score of 15 [13] and a precision of 1.8 points to give a sample size of 267. To account for nonresponse, the sample size was inflated by 15% to give a total sample size of 308 respondents at both sites. The sample was split between the two facilities based on the total number of health workers in each facility.

The healthcare workers were selected by department. In the first step, a list of departments and health workers deployed to the departments was generated. The number of participants in each department was then apportioned from the sample size in the facility based on the proportion of workers in the department relative to the health worker population. In each department, healthcare workers were randomly selected to participate in the study.

### 2.3. Data Collection Methods

Data were collected using a self-administered structured questionnaire. The questionnaire was grouped into four parts. The first part collected data on sociodemographic variables of the participants such as sex, age, marital status, and duration of practice.

Part two collected data to assess the knowledge of depression. Questions that had been used in other studies and the WHO Mental Health Gap Action Programme were used to select items for the knowledge and practice sections [7]. The knowledge component had a total of 14 questions, using a yes/no/do not know format. The first 10 questions were applicable to all HCWs and covered aspects of depression, such as general awareness, misconceptions, suicide risk, and treatment options. For healthcare workers whose routine duties included diagnosis and treatment of depression (doctors, clinical officers, nurses, and pharmacists), four knowledge questions on drugs for depression were included.

In part three, data on attitudes towards depression were collected by applying the Revised Depression Attitude Questionnaire (R-DAQ) to assess the attitudes of participants towards depression. R-DAQ is a tool that measures participants' attitudes towards depression through a 22-item scale [14]. The R-DAQ looks at three broad factors (subscales) in assessing attitude: the perspective of health workers on the occurrence of depression, its management, and recognition; optimism about the treatment for depression; and confidence in the care of depression. The R-DAQ allows comparison of health workers' attitudes across cultures and regions because, together with its previous version, it has been applied in countries on different continents.

Part four of the questionnaire included 16 questions on healthcare workers' practices for depression. The questions ranged from the frequency of depression screening to personal attitudes towards time constraints in managing depression and choices in patient referrals. We also explored the availability of mental health services in the facilities and the training received. The questions were a mix of multiple-choice, yes/no, and Likert scale formats.

### 2.4. Data Analysis

Statistical analysis was conducted using the R statistical software [15]. Participants' characteristics were described using frequencies for categorical variables and medians and interquartile ranges for continuous variables. The associations between sociodemographic variables and various measures of knowledge and attitudes were compared using chi-square tests, or Fisher's exact tests as appropriate.

To determine the knowledge score, each correct response to the knowledge statements was coded as 1, with an incorrect statement response recorded as 0. These were then summed to give the knowledge score for each participant, with a maximum of 10 and a minimum of zero. A higher score indicated greater knowledge of depression, while a lower score indicated gaps in knowledge.

The attitude statements had a five-level Likert scale. To determine the attitude score, a response indicating a good attitude was coded as 5, while that showing a poor attitude was coded as 1. These were then summed to give the attitude score a maximum of 110 and a minimum of 22. A higher score indicated a more optimistic view of depression and its management.

Univariable and multivariable regression was conducted separately for knowledge and attitude scores against selected factors such as years practiced, professional cadre, marital status, gender, work department, and reported chronic illness. HCWs were categorised into three for purposes of analysis: nurses, clinicians (doctors/clinical officers), and nonclinicians (other healthcare workers). Independent variables whose *p* values were less than 0.2 in the univariable analysis were included in the multivariable linear regression model. Variable selection was done through the backward elimination method, while model selection was based on Akaike information criteria [16]. A *p* value of < 0.05 was considered statistically significant.

### 2.5. Ethical Considerations

Written informed consent was obtained from all participants, and the study was reviewed and approved by the USIU-Africa Institutional Review Board (USIU-A/IRB/370-2021) and the Kijabe Hospital IRB (KH/IERC/02718/0117/2022). Administrative approval was sought from the participating facilities, and a research permit was obtained from the National Commission for Science, Technology, and Innovation. Only the first author had access to the signed consent forms, and the other authors did not have access to identifiable data.

## 3. Results

### 3.1. Sociodemographic and Work-Related Characteristics

**Figure 1:**
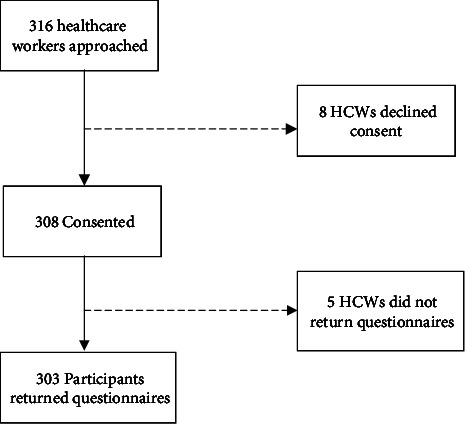
Flow chart of study enrolment.

A total of 316 healthcare workers were approached, and 303 (95.9%) consented and participated in the study (Figure 1). Eight potential participants declined consent, and five did not return the questionnaire.

The majority (59.4%) of the respondents were from Kijabe Hospital. And nearly two-thirds (64.0%) were female. The majority (58.4%) of the respondents were between 18 and 29 years old, with only 8% aged 50 years and above. Only 5.3% of the respondents reported having a chronic illness. The chronic illnesses reported included diabetes, hypertension, asthma, and heart disease. The most common professional cadre was nurse (40.6%), and the majority (53.8%) of the respondents had less than 5 years of professional practice (Table 1). Most of the respondents worked in medical wards (24.1%) and outpatient clinics (20.5%), while 29.7% worked in pediatric or obstetric wards (Table 1).

### 3.2. Knowledge of Depression among Healthcare Workers

Almost all (>90%) of the respondents had heard of depression, considered depression a health problem, knew that depressed patients can break down at any time, and said that depression can lead to suicide or suicide attempts or that depression can be treated with pharmacological methods and psychotherapy. Similarly, nearly all respondents (>90%) correctly disagreed that depression is caused by witchcraft or that depression is best managed by traditional healers. About 10% of the respondents wrongly agreed that depression is a disease of a particular age group or that depressed patients are dangerous to themselves and others (Supplementary Table 1).

Respondents (*n* = 227) involved in the diagnosis and medical treatment (doctors, clinical officers, and pharmacists) of depression were asked about drugs used in depression. Only three-quarters (76.2%) of the respondents knew that fluoxetine is an antidepressant (fluoxetine is the antidepressant of choice at the primary care level). Almost half of the respondents (44.1%) wrongly agreed that carbamazepine is an antidepressant. (Supplementary Table 1).

From a possible maximum of 10, the median knowledge score was 9 (interquartile range (IQR): 8-9). In the univariable analysis, the professional cadre, work department, and study hospital were statistically associated with knowledge scores. Nonclinician staff, on average, scored 0.49 points lower on the knowledge score compared to clinicians (*p* < 0.001). (Table 2).

In multivariable linear regression, the professional cadre was independently associated with the knowledge score, while the other variables were not statistically significant. Nonclinicians had significantly lower (*β* = −0.5, *p* = 0.011) knowledge scores compared to clinicians.

### 3.3. Attitudes towards Depression among Healthcare Workers

In general, attitudes towards statements under the professional confidence in professional care and therapeutic optimism to depression subscales were mostly positive. Attitudes were mostly negative for statements under the general perspectives about the occurrence and management subscale of depression.

Although 46.2% of the respondents strongly agreed or agreed that they were comfortable dealing with depressed patients' needs, only 9.3% strongly agreed or agreed that they were confident in assessing the risk of suicide in patients with depression. Two-thirds and nine-tenths of the respondents strongly agreed or agreed that depression was a natural part of adolescence or that it is a response that is not amenable or agreed that all health professionals should have the skills to recognise and manage depression or that managing depression is an important part of managing other health problems (Supplementary Table 2).

**Figure 2:**
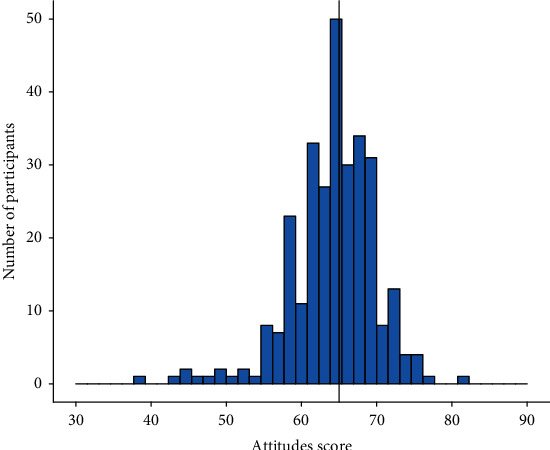
Attitude score on depression among respondents. The median is indicated by a solid vertical line.

The median attitude score was 65 (IQR 62-68). The minimum and maximum scores were 38 and 81, respectively (Figure 2).

On multivariable linear regression with attitude score as a dependent variable, the knowledge score was positively associated with attitude score, with every unit increase in knowledge score associated with a 0.78 increase in attitude score (*p* = 0.009). Similarly, respondents with professional experience of 5-14 years had higher attitude scores (*β* = 1.7, *p* = 0.023) compared to those with professional experience of fewer than 5 years (Table 3).

### 3.4. Practice in the Care of Depression among Healthcare Workers

Almost half (47%) of the respondents reported that they rarely or never screened patients for depression. Among clinicians and nurses, 40.3% reported that they rarely or have never screened for depression and half reported only screening patients for depression occasionally. Although two-thirds of the respondents would refer depressed patients for counseling, only 12% would primarily consider medical management for depression. Only one-third (34%) reported receiving formal training in mental health after their basic training, with 61.5% attending the training for four weeks or less.

## 4. Discussion

We found that HCW had good knowledge about the etiology and symptoms of depression, but their understanding of its pharmacological management was less robust. Nonclinical staff generally had lower knowledge scores than clinical staff. Attitudes towards depression were mostly positive, especially regarding professional confidence in care and therapeutic optimism. However, they were more negative in general perspectives about its occurrence and management. Knowledge levels positively influenced attitudes, with more experienced professionals showing more positive attitudes. Half of the respondents seldom screened for depression, and only a minority recommended comprehensive management approaches.

Nonclinical staff exhibited notably lower levels of knowledge compared to clinical staff, including doctors and clinical officers. However, the difference in knowledge scores, while significant, was relatively small in magnitude. Regarding attitudes towards depression, individuals who had worked for 5-14 years displayed higher attitude scores compared to those who had worked for less than 5 years. Moreover, knowledge scores were positively correlated with attitude scores, indicating that possessing specific knowledge about depression influences the attitudes of healthcare workers (HCWs) towards it [17]. It can be inferred that HCWs with longer work experience were more likely to have encountered patients with depression, gained more knowledge, and consequently developed more positive attitudes towards the condition. We found that healthcare workers had a modest to good understanding of depression as a mental illness. Most HCWs had high levels of knowledge of depression management, including predisposing factors, causes, symptoms, and treatment. Additionally, the findings of this study demonstrated that healthcare workers were aware that patients with depression are a risk to themselves and others, as well as the fact that depression can be treated using pharmacological and psychotherapy treatments.

The knowledge levels reported in our study were generally higher than those reported in similar studies in Kenya, Tanzania, Zambia, and Nigeria, which were all conducted at least five years before the current study [18–20]. Higher levels of knowledge about depression in this study could reflect increased awareness of depression and mental health in general on the backdrop of the COVID-19 pandemic [21]. In the past five years, there has been an increased focus on depression, including the Ministry of Health launching a Mental Health Action Plan in 2021 [8] and the WHO having a dedicated World Health Day on depression in 2017 [22].

An area of low awareness among respondents was the treatment of depression and the use of medication. This finding is consistent with studies from Nigeria and Cameroon that reported that most medical personnel have little or no training in treatment protocols for mental health disorders [20, 23]. Our study found that less than half of the respondents felt comfortable dealing with depression, although the majority were confident in addressing depression in patients. Respondents had favourable attitudes towards the effects of psychotherapy and antidepressant drugs. However, when asked about a specific aspect of depression in assessing suicide risk, less than 10% had confidence in handling suicide risk. The low awareness of depression treatment and medication use among respondents could lead to inadequate care for patients with depression, particularly in severe cases like suicide risk.

Our findings on attitudes towards depression were similar to those on knowledge, where participants showed a high awareness of general aspects of depression but less so when asked about specific aspects of depression. While most respondents felt that their profession had a role to play in the care of depressed patients, fewer were confident in the specific treatment of depressed patients. Similar findings of health professionals who are mostly not comfortable managing depression patients have been reported in studies in Nigeria, Cameroon, and Kenya [18, 20, 23]. The less positive attitudes towards mental illness and depression could be due to inadequate training of health workers which makes them less confident to handle patients with depression and other mental illnesses. Most of the participants reported the need for additional training in mental health.

Despite positive attitudes towards professional confidence in professional care, there were more negative attitudes towards some of the risk factors and associations with depression. The majority of those who participated in the survey believed that clinical depression is either an unavoidable characteristic of the adolescent years or a reaction that cannot be altered. More than a quarter of those who participated in the survey had unfavorable attitudes towards depression, believing that the condition was caused by a lack of self-discipline and willpower, that it was an inevitable aspect of getting older, and that suicidal ideation could not be prevented. These negative attitudes likely reflect low knowledge of specific aspects of depression management, and it is likely that most HCW knowledge is based on nonspecific information acquired from interactions with colleagues or nonmedical literature. Furthermore, some studies have shown that knowing about mental illness does not necessarily translate to a positive attitude towards mental illness because myths and social stigma at times overrun one's knowledge [24]. In African society, mental illness is often associated with witchcraft and other negative myths, which likely contribute to healthcare workers' attitudes [18].

Attitudes were lowest on depression occurrence and management. Less than 20% of those who participated in the survey believed that people who suffer from depression have care requirements that are comparable to those who suffer from other chronic illnesses or that the treatment of depression is an essential component of the management of other health problems. This finding of a negative attitude towards depression management and outcome is probably because healthcare personnel do not commonly interact with depressed patients. Negative attitudes are obstacles to the provision of mental healthcare, which can have ramifications such as delays in treatment, high levels of recurrence, and other nonscientific treatment modalities, ultimately leading to an increase in the prevalence of mental diseases such as depression [18, 25].

Four-fifths of clinicians and nurses did not routinely screen patients for depression, one of the most common causes of healthcare visits. This likely subjected patients to medium- and longer-term risks associated with depression, including suicide due to late detection.

The study had several limitations. We conducted the study in two middle-sized facilities that may not be representative of the breadth of facilities and healthcare workers in the country. This study applied a survey design that may not allow for a conclusive investigation of the reasons for some of the practices and attitudes. A qualitative study involving focus group discussions could provide insights that can inform training interventions.

In conclusion, the study found that HCWs generally had good knowledge of depression's etiology and symptoms but were less confident in its pharmacological management. This was especially true for nonclinicians and staff with fewer years of experience. While attitudes towards depression care were mostly positive, gaps exist in regular screening and comprehensive management practices. Enhancing training, for both clinical and nonclinical staff and younger staff, could lead to improved early detection and treatment.

## Figures and Tables

**Table 1:** Sociodemographic and other characteristics of the respondents from two medium-sized hospitals in Kenya, 2022.

Characteristic	Number	Proportion	
		%	95% CI
Hospital			
Kijabe	180	59.4	53.6-64.9
Naivasha	123	40.6	35.1-46.4
Sex			
Female	194	64.0	58.3-69.4
Male	109	36.0	30.6-41.7
Age group (years)			
18-24	48	15.8	12.0-20.6
25-29	129	42.6	37.0-48.4
30-34	56	18.5	14.4-23.4
35-39	20	6.6	4.2-10.2
40-49	26	8.6	5.8-12.5
50-59	22	7.3	4.7-10.9
≥60	2	0.7	0.1-2.6
Reported chronic illness	16	5.3	3.1-8.6
Professional cadre			
Nurse	123	40.6	35.1-46.4
Clinical officer	61	20.1	15.9-25.2
Lab technologist	28	9.2	6.3-13.2
Doctor	23	7.6	5.0-11.3
Pharmacist	20	6.6	4.2-10.2
Physiotherapy	10	3.3	1.7-6.2
Dental staff	8	2.6	1.2-5.3
Other⁣^∗^	30	9.9	6.9-14
Years worked as health worker			
<1	64	21.1	16.8-26.2
1-4	99	32.7	27.5-38.3
5-9	29	9.6	6.6-13.6
10-14	69	22.8	18.3-28
15-19	10	3.3	1.7-6.2
20-24	13	4.3	2.4-7.4
25-29	8	2.6	1.2-5.3
≥30	11	3.6	1.9-6.6
Current department			
Medical ward	73	24.1	19.5-29.4
Outpatient clinics	62	20.5	16.2-25.5
Pediatric ward	49	16.2	12.3-20.9
Obstetrics ward	41	13.5	10.0-18.0
Surgical ward	31	10.2	7.2-14.3
Laboratory	28	9.2	6.3-13.2
Emergency	14	4.6	2.6-7.8
Other⁣^∗∗^	5	1.7	0.6-4.0

⁣^∗^Counsellor, psychologist, occupational therapist, nutritionist, and public health officer. ⁣^∗∗^Administration.

**Table 2:** Univariable and multivariable linear regression of the knowledge score on depression against covariates in two medium-sized hospitals in Kenya, 2022.

Characteristic	Univariable analysis			Multivariable analysis		
	Beta	95% CI	*p* value	Beta	95% CI	*p* value
Professional cadre						
Clinician	Ref	Ref		Ref	Ref	
Nonclinician	-0.49	-0.82, -0.17	0.003	-0.50	-0.88, -0.12	0.011
Nurse	-0.03	-0.34, 0.27	0.8	-0.03	-0.34, 0.27	0.8
Work department						
Outpatient	Ref	Ref		Ref	Ref	
Inpatient	0.26	0.00, 0.53	0.052	-0.01	-0.34, 0.31	>0.9
Work years as healthcare worker⁣^∗^						
<5	Ref	Ref		—	—	—
5-14	0.10	-0.18, 0.39	0.5	—	—	—
15+	0.17	-0.22, 0.56	0.4	—	—	—
Marital status⁣^∗^						
Single	Ref	Ref		—	—	—
Married	0.09	-0.17, 0.34	0.5	—	—	—
Hospital⁣^∗^						
Kijabe	Ref	Ref		—	—	—
Naivasha	0.14	-0.12, 0.40	0.3	—	—	—
Sex⁣^∗^						
Female	Ref	Ref		—	—	—
Male	-0.13	-0.40, 0.13	0.3	—	—	—
Reported chronic disease⁣^∗^						
No	Ref	Ref		—	—	—
Yes	-0.15	-0.73, 0.43	0.6	—	—	—

Ref: reference category; CI: confidence interval. ⁣^∗^Not included in the multivariable model.

**Table 3:** Univariable and multivariable linear regression of the attitude score on depression against various covariates from two medium-sized hospitals in Kenya, 2022.

Characteristic	Univariable analysis			Multivariable analysis		
	Beta	95% CI	*p* value	Beta	95% CI	*p* value
Knowledge score	0.69	0.12, 1.3	0.019	0.78	0.20, 1.4	**0.009**
Years worked as a healthcare worker						
<5 yrs	Ref	Ref		Ref	Ref	
5-14 yrs	1.3	-0.16, 2.7	0.083	1.7	0.23, 3.2	**0.023**
15+ yrs	0.59	-1.4, 2.6	0.6	1.4	-0.66, 3.6	0.2
Professional cadre						
Clinician	Ref	Ref		Ref	Ref	
Nonclinician	0.49	-1.2, 2.2	0.6	1.3	-0.66, 3.3	0.2
Nurse	-1.2	-2.8, 0.33	0.12	-1.6	-3.2, 0.04	0.056
Work department						
Outpatient	Ref	Ref		Ref	Ref	
Inpatient	-0.79	-2.1, 0.56	0.2	0.51	-1.2, 2.2	0.6
Hospital						
Kijabe	Ref	Ref				
Naivasha	0.78	-0.54, 2.1	0.2	1.1	-0.22, 2.5	0.10
Sex⁣^∗^						
Female	Ref	Ref		--	--	--
Male	0.14	-1.2, 1.5	0.8	--	--	--
Marital status⁣^∗^						
Single	Ref	Ref		--	--	--
Married	0.42	-0.89, 1.7	0.5	--	--	--
Reported chronic disease⁣^∗^						
No	Ref	Ref		--	--	--
Yes	1.2	-1.8, 4.1	0.4	--	--	--

Ref: reference category; CI: confidence interval. ⁣^∗^Not included in multivariable model. Significant *p* values are indicated in bold.

## Data Availability

The datasets used and/or analysed during the current study are available from the corresponding author on reasonable request.

## Abbreviations

AIC: Africa Inland Church
COVID-19: Coronavirus disease 2019
HCP: Healthcare providers
HCW: Healthcare workers
IRB: Institutional Review Board
IQR: Interquartile range
KAP: Knowledge, attitudes, and practices
MDD: Major depression disorder
R-DAQ: Revised Depression Attitude Questionnaire
USIU-A: United States International University-Africa
WHO: World Health Organization.

## Supplementary Materials

Supplementary Materials Supplementary Table 1: knowledge of depression among healthcare workers, 2022. Supplementary Table 2: attitudes to depression among healthcare workers, 2022.
