# Supplementary material for: Knowledge, Attitudes, and Practices among Healthcare Workers regarding Depression Care in Two Medium-Sized Hospitals in Kenya
Source: Depress Anxiety. 2024 Jun 7;2024:4756962. doi: 10.1155/2024/4756962 (PMC11918951; doi:10.1155/2024/4756962)
Supplement: Supplementary Materials — Supplementary Table 1: knowledge of depression among healthcare workers, 2022. Supplementary Table 2: attitudes to depression among healthcare workers, 2022. [file 4756962.f1.docx]

**Supplementary Table 1: Knowledge of Depression among Healthcare workers, 2022**

| **Statement** | **Agree**  **n (%)** | **Disagree**  **n (%)** | **Don’t Know n (%)** | **No Response**  **n (%)** |
| --- | --- | --- | --- | --- |
| Have you ever heard about depression? | 297 (98.0) | 3 (1.0) | 2 (0.7) | 1 (0.3) |
| Do you consider depression as a health problem? | 294 (97.0) | 4 (1.3) | 0 (0.0) | 5 (1.7) |
| Depression affects people of a particular age group | 36 (11.9) | 255 (84.2) | 6 (2.0) | 6 (2.0) |
| Depression is caused by witchcraft, charms, evil spirits | 9 (3.0) | 280 (92.4) | 8 (2.6) | 6 (2.0) |
| Patients with depression can breakdown at anytime | 275 (90.8) | 13 (4.3) | 9 (3.0) | 6 (2.0) |
| Patients with depression are dangerous to themselves and others | 266 (87.8) | 27 (8.9) | 6 (2.0) | 4 (1.3) |
| Depression can lead to suicide or suicide attempts | 297 (98.0) | 2 (0.7) | 0 (0.0) | 4 (1.3) |
| Depression can be treated with pharmacological methods and psychotherapy | 279 (92.1) | 12 (4.0) | 8 (2.6) | 4 (1.3) |
| Depression is best managed by traditional doctors/healers | 1 (0.3) | 278 (91.7) | 15 (5.0) | 9 (3.0) |
| Depression responds better to traditional remedies than orthodox treatment most of the time | 5 (1.7) | 253 (83.5) | 42 (13.9) | 3 (1.0) |
| Amitriptyline is an anti-depressant drug* | 212 (93.4) | 6 (2.6) | 5 (2.2) | 4 (1.8) |
| Methotrexate is an anti-depressant drug* | 34 (15.0) | 158 (69.6) | 24 (10.6) | 11 (4.8) |
| Fluoxetine is an anti-depressant drug* | 173 (76.2) | 23 (10.1) | 22 (9.7) | 9 (4.0) |
| Carbamazepine is an anti-depressant drug* | 100 (44.1) | 107 (47.1) | 14 (6.2) | 6 (2.6) |
| *Question only asked to nurses, doctors, clinical officers and pharmacists (n=227) | | | | |

**Supplementary Table 2: Attitudes to Depression among Healthcare workers, 2022**

| **Statement** | **Strongly agree n**  **(%)** | **Agree  n**  **(%)** | **Neutral n**  **(%)** | **Disagree n**  **(%)** | **Strongly disagree n**  **(%)** |
| --- | --- | --- | --- | --- | --- |
| **Professional confidence in professional care** |  |  |  |  |  |
| I feel comfortable in dealing with depressed patients’ needs | 27  (8.9) | 113 (37.3) | 85 (28.1) | 60  (19.8) | 9  (3.0) |
| I feel confident in assessing depression in patients | 147 (48.5) | 112 (37.0) | 10  (3.3) | 11  (3.6) | 15  (5.0) |
| I am more comfortable working with physical illness than with mental illnesses like depression | 56  (18.5) | 105 (34.7) | 59 (19.5) | 53  (17.5) | 19  (6.3) |
| My profession is well placed to assist patients with depression | 91  (30.0) | 110 (36.3) | 25 (8.3) | 39  (12.9) | 30  (9.9) |
| My profession is well trained to assist patients with depression | 51  (16.8) | 129 (42.6) | 60 (19.8) | 40  (13.2) | 14  (4.6) |
| I feel confident in assessing suicide risk in patients presenting with depression | 6  (2.0) | 22  (7.3) | 32 (10.6) | 135 (44.6) | 98  (32.3) |
| It is rewarding to spend time looking after depressed patients | 32  (10.6) | 112 (37.0) | 78 (25.7) | 55  (18.2) | 15  (5.0) |
| **Therapeutic optimism to depression** |  |  |  |  |  |
| Psychological therapy tends to be unsuccessful with people who are depressed | 11  (3.6) | 21  (6.9) | 23 (7.6) | 147 (48.5) | 88  (29.0) |
| Antidepressant therapy tends to be unsuccessful with people who are depressed | 3  (1.0) | 10  (3.3) | 48 (15.8) | 138 (45.5) | 93  (30.7) |
| One of the main causes of depression is a lack of self-discipline and will-power | 22  (7.3) | 55 (18.2) | 37 (12.2) | 95  (31.4) | 80  (26.4) |
| Depression treatments medicalise unhappiness | 24  (7.9) | 133 (43.9) | 77 (25.4) | 46  (15.2) | 12  (4.0) |
| Becoming depressed is a natural part of being old | 22  (7.3) | 63 (20.8) | 32 (10.6) | 102 (33.7) | 65  (21.5) |
| Becoming depressed is a way that people with poor stamina deal with life difficulties | 32  (10.6) | 107 (35.3) | 57 (18.8) | 79  (26.1) | 18  (5.9) |
| Once a person has made up their mind about taking their own life no one can stop them | 29  (9.6) | 86 (28.4) | 66 (21.8) | 84 (27.7) | 29  (9.6) |
| Depression reflects a response which is not amenable to change | 167 (55.1) | 108 (35.6) | 6  (2.0) | 3  (1.0) | 10  (3.3) |
| Becoming depressed is a natural part of adolescence | 87  (28.7) | 119 (39.3) | 18  (5.9) | 40  (13.2) | 25  (8.3) |
| There is little to be offered to depressed patients who do not respond to initial treatments | 7  (2.3) | 20  (6.6) | 25  (8.3) | 134 (44.2) | 104 (34.3) |
| **General perspective about depression occurrence and management** | | | |  |  |
| Depression is a disease like any other (e.g. asthma, diabetes) | 78  (25.7) | 128 (42.2) | 14  (4.6) | 45  (14.9) | 28  (9.2) |
| All health professionals should have skills in recognizing and managing depression | 13  (4.3) | 8  (2.6) | 18  (5.9) | 103 (34.0) | 153 (50.5) |
| People with depression have care needs similar to other medical conditions like diabetes, COPD or arthritis | 8  (2.6) | 42 (13.9) | 32 (10.6) | 142 (46.9) | 66  (21.8) |
| Recognizing and managing depression is often an important part of managing other health problems | 8  (2.6) | 22 (7.3) | 40 (13.2) | 113 (37.3) | 110 (36.3) |
| Anyone can suffer from depression | 102 (33.7) | 139 (45.9) | 35 (11.6) | 9  (3.0) | 2  (0.7) |
